# Impact of rumen microbiome sample handling and extraction procedures on downstream long-read sequencing

**DOI:** 10.3389/fcimb.2026.1907783

**Published:** 2026-10-28

**Authors:** Rocio Amorin de Hegedus, Kevin Panke-Buisse, Thaddeus C. Deiss, Jennifer C. McClure

**Affiliations:** 1 US Dairy Forage Research Center, Agricultural Research Service, United States Department of Agriculture (USDA), Madison, WI, United States; 2 Oak Ridge Institute for Science and Education, Oak Ridge, TN, United States

**Keywords:** DNA integrity, long-read sequencing, microbiome, rumen, sample preservation

## Abstract

The rumen microbiome is a complex microbial ecosystem central to ruminant nutrition and productivity. Long-read sequencing technologies, such as Oxford Nanopore, have greatly improved resolution of strain-level diversity and enabled assembly of near-complete genomes, but these benefits depend on the integrity and purity of input DNA, which can be affected by sample preservation and extraction methods. This study evaluated multiple DNA extraction protocols to best maximize high–molecular-weight DNA recovery for long-read sequencing and whether flash freezing or freezing and thawing samples biased read length, microbial composition, or the recovery of different taxa. We also used simulated datasets to assess how read length influences taxonomic classification accuracy. Rumen fluid samples of equal volume from four lactating Holstein cows were preserved using two different methods: 1. Pelleting 1 ml of rumen fluid then flash freezing; and 2. bulk freezing 15ml aliquots, thawing, then pelleting 1 ml of rumen fluid. DNA was extracted using four modified Zymo Quick-DNA HMW MagBead protocols (0BB, 3BB, DDZ, PREC) incorporating different lysis and purification strategies, then sequenced on Oxford Nanopore PromethION flow cells. Read length, yield, and microbial composition were analyzed using nonparametric statistics, while synthetic long-read datasets (400–7600 bp) generated with Nano Sim evaluated classification accuracy with Kaiju. The precipitation method produced the highest DNA yields and maintained fragment integrity, whereas bead-based methods yielded less DNA due to high–molecular-weight DNA–selective binding. Preservation of DNA quality was identified in FF over FT samples with longer reads and higher archaeal and Gram-positive abundances. Read lengths were reduced in FT samples for both Gram-positive and Gram-negative taxa, with Gram-negative bacteria showing the greatest loss. Simulations confirmed that longer reads improve taxonomic classification, underscoring the need to optimize upstream protocols for high-resolution rumen microbiome profiling.

## 1 Introduction

The rumen microbiome is a complex and highly diverse microbial ecosystem composed of bacteria, archaea, protozoa, and fungi that collectively play a central role in feed digestion and animal performance (Huws et al., 2018). Understanding the composition and functions of this community is essential for improving livestock production. However, the study of this community has historically been constrained by technological bottlenecks, with progress occurring through advances in sequencing methods. Early work based on cultivation and short-read amplicon sequencing provided only a coarse view of microbial diversity and often failed to resolve species or strain-level variation (Johnson et al., 2019). Furthermore, current extraction procedures rely either on long mechanical lysis steps or chemical lysis, which cannot account for differences in cellular integrity found in different microbial populations (Henderson et al., 2013; Yu and Morrison, 2004). Different lysis methods may lead to longer fragments from more robust microbes like gram-positive bacteria, and shorter fragments from those that are less robust like gram-negative bacteria.

Advances in long-read sequencing technologies, particularly Oxford Nanopore, have transformed our ability to study microbial communities by enabling the assembly of near-complete genomes directly from complex metagenomes (Liu et al., 2022; Cusco et al., 2021). Compared to short-read approaches, long reads resolve repetitive regions, structural variants, and strain-level diversity with much greater accuracy, providing deeper insight into community composition and genome architecture (Chen et al., 2022). However, the quality of metagenomic assemblies generated from long reads is highly dependent on the integrity and purity of input DNA (Schalamun et al., 2019; Trigodet et al., 2022), making sample preservation and extraction methods critical to downstream results.

DNA preservation strategies, such as flash-freezing (FF) or freeze–thaw (FT) cycles can significantly affect the structural integrity of microbial cells. This occurs through ice crystal formation and osmotic stress as water inside and around cells freezes (El-Kest and Marth, 1992). Gram-negative bacteria are particularly prone to lysis under these conditions which can lead to DNA degradation and selective loss of these taxa, thus impacting the observed microbial composition and metagenomic assembly. Addressing these gaps will help refine preservation strategies, ensuring that they do not compromise the accuracy of microbial diversity analyses.

Furthermore, the DNA extraction method plays a critical role in determining the yield and integrity of the obtained genetic material. This is especially important in the context of long-read sequencing, which relies on the recovery of high molecular weight (HMW) DNA to produce accurate and complete results. Phenol chloroform extraction has historically been considered the gold standard for obtaining large quantities of high-quality DNA from challenging samples (Molbert et al., 2023; Gautam, 2022; Köchl et al., 2005). However, its drawbacks include labor-intensive protocols, reduced yield when paired with size selection (Maghini et al., 2021), increased co-extraction of host DNA from certain sample types (Trigodet et al., 2022), and the volatility and toxicity of the chemicals involved (Barbier et al., 2019; Mott et al., 2022). These limitations have encouraged the search for alternative approaches. For instance, Cusco et al. (2021) successfully recovered complete bacterial genomes from canine feces using the Zymo Quick-DNA HMW MagBead kit, and Gand et al. (2023) found the same kit to produce the highest yields among six tested methods for HMW bacterial DNA. Importantly, Mott et al. (2022) also demonstrated that extraction methods and bead-beating intensity could shift the observed community composition in bovine rumen and fecal samples, even altering the size distribution of recovered DNA. Building on this prior work, we further tested modifications of the Zymo Quick-DNA HMW MagBead kit on rumen samples to optimize yield and improve recovery of recalcitrant microbial taxa.

In this study, we evaluate the effects of sample preservation and extraction methods on metagenomic outcomes. Specifically, we address two key research questions: 1) What is the best DNA extraction method to balance the recovery of HMW DNA across microbial populations, and 2) Does sample treatment prior to extraction introduce biases in read length, microbial representation, or gram-positive versus gram-negative proportions? To answer the first question, we assess extraction performance in terms of total yield, read length distribution, and taxonomic breadth, focusing on whether certain methods disproportionately favor or exclude specific microbial populations. For the second, we test the hypothesis that a single freeze-thaw cycle may reduce the recovery of gram-negative taxa due to increased cell fragility, leading to changes in community structure. Together, these questions help inform how preprocessing steps impact downstream data quality and posterior microbial analysis.

## 2 Materials and methods

### 2.1 Simulated read generation and taxonomic classification

To investigate how read length influences taxonomic classification accuracy, we generated synthetic long-read datasets using Nano Sim (v3.0) based on a curated set of 22 rumen-associated microbial genomes (Yang et al, 2017). Genomes were selected to represent a broad phylogenetic diversity within the rumen microbiome, including bacteria and archaea. The genome set included: *Bifidobacterium animalis*, *Butyrivibrio fibrosolvens*, *Clostridium cellulolyticum*, *Eubacterium cellulosolvens*, *Eubacterium ruminantium*, *Megasphaera elsdenii*, *Methanobacteriaceae archaeon*, *Ruminococcus albus*, *Ruminococcus flavefaciens*, *Segatella albensis*, *Succinivibrio dextrinosolvens*, *Xylanibacter ruminicola*, *Prevotella ruminicola*, *Fibrobacter succinogenes*, *Lachnospira multipara*, *Methanobrevibacter ruminantium*, *Selenomonas ruminantium*, *Succinimonas amylolytica*, *Methanosarcina barkeri*, *Treponema bryantii*, *Selenomonas bovis*, and *Klebsiella pneumoniae*.

Using Nano Sim’s *simulator.py* script in metagenome mode, we simulated reads for 12 distinct length bins: 400–600 bp, 600–800 bp, 800–1000 bp, 1000–1200 bp, 1200–1400 bp, 1400–1600 bp, 2400–2600 bp, 3400–3600 bp, 4400–4600 bp, 5400–5600 bp, 6400–6600 bp, and 7400–7600 bp. For each length bin, 20,000 reads were generated using default simulation parameters. Simulated reads were classified using Kaiju (v1.9.2) with the *-m 12* and *-a mem* options, against the nr_euk reference database (Menzel et al, 2016). The Kaiju output was parsed to extract the assigned taxonomy, and each read was labeled as Correct, Misclassified, or Unclassified at Genus Level by comparing the assigned genus to the known genus of origin.

### 2.2 Sample collection

Rumen fluid collection was approved by the University of Wisconsin Institutional Animal Care and Use Committee (protocol A006391-R01). Approximately 100 mL of rumen contents were collected from the cannula of multiparous lactating Holstein cows at 212 and 232 days in milk (DIM). The procedure was repeated with two additional multiparous lactating Holstein cows at 452 and 129 DIM to validate the reproducibility of the findings. All cows were fed a total mixed ration primarily composed of corn and alfalfa silage. At the time of sampling, rumen contents were strained cow-side through four layers of cheesecloth to separate the liquid fraction from the solids. The liquid portion was immediately transferred into airtight containers and transported to the laboratory within 30 minutes of collection.

### 2.3 Preservation method

The standard method of rumen collection for nucleic acid extraction involves bulk freezing rumen fluid aliquots (Henderson et al., 2013; Fliegerova et al., 2014; Mott et al, 2022). To test the impact of preservation on DNA quality, two strategies were applied. Fifteen milliliters of each sample was aliquoted into a 15 mL conical tube and bulk frozen at −80 °C. An additional 9 mL was divided into 1 mL aliquots, centrifuged at 17,000 × *g* for 10 minutes to pellet microbial cells, the supernatant was discarded and the resulting pellets were flash frozen in liquid nitrogen before being stored at −80 °C (designated as flash-frozen, FF). After 30 days, the 15 mL rumen liquid tubes were thawed at room temperature for approximately one hour, mixed thoroughly by inversion, and divided into nine 1 mL was aliquots. These were centrifuged at 17,000 × *g* for 10 minutes, supernatant was removed, and the pellet was flash frozen in liquid nitrogen and stored at −80 °C. These samples were labeled as freeze-thaw (FT). This approach ensured uniform input volume and minimized the number of freeze-thaw cycles across all treatments (**Supplementary Figure 1**).

### 2.4 DNA extraction methods

To mimic what is typically found in the literature, DNA was extracted from bulk frozen samples using four modified protocols based on the Zymo Quick-DNA HMW MagBead kit (Zymo Research, Irvine, CA). This kit was selected for its promising performance in a previous study (Unpublished data). All pelleted samples were thawed at room temperature in 200 μL of DNA/RNA Shield nucleic acid preservative (Zymo Research). To ensure adequate incorporation the pellets were periodically stirred with a pipette tip to fully incorporate the preservative. Once thawed, DNA/RNA Shield was removed following the manufacturer’s protocol and set aside for reincorporation later. A two-step enzymatic digestion was performed across all methods at different stages of extraction while 2 methods, DDZ and PREC, repeated the digestion following mechanical lysis. The first step involved incubation for 30 minutes at 37 °C with 25 μL of lysozyme (100 mg/mL; Sigma, St. Louis, MO). After lysozyme digestion, the preservative treated supernatant was returned to the tube, and a second digestion was carried out by incubating the samples with 40 μL of 10% SD S and 20 μL of Proteinase K at 55 °C for 20 minutes. DNA was then purified using either 40 ul of Zymo magnetic beads or ethanol precipitation depending on the extraction group. Group 1 (0BB) is outlined above, without a bead beating step. DNA was eluted from magnetic beads in 75 μL. Group 2 (3BB) samples underwent three minutes of mechanical lysis via bead beating in DNA/RNA Shield, followed by PB S washing according to Zymo’s protocol. The pellet was then subjected to enzymatic digestion, and the DNA was purified via magnetic beads and eluted in 75 μL of Zymo Elution Buffer.

Groups 3 and 4, double digestion followed by Zymo magnetic bead extraction (DDZ), and double digestion followed by ethanol precipitation (PREC), began like 0BB with a two-step enzymatic digestion and centrifugation at 8,000 × *g* for one minute. The supernatant was collected and placed on ice. Afterwards, the residual pellet was beaten for three minutes at 2400 rpm in a Biospec Mini BeadBeater 24 disruptor (Bartlesville, OK U SA) and subjected to a second two-step enzymatic digestion. The sample was then centrifuged at 8,000 xg for one minute, and the supernatants from digestion 1 and digestion 2 were combined before magnetic bead purification or ethanol precipitation.

DDZ samples were purified from the combined supernants using magnetic beads, following the standard Zymo protocol, and eluted in 75 μL. For PREC, two volumes of magbead binding buffer (Zymo Research) were added to the combined supernants, followed by three volumes of ethanol to precipitate DNA. Samples were centrifuged at 8,000 × *g* for 10 minutes at 4 °C, and the DNA pellet was washed twice with 70% ethanol, air-dried, and resuspended in > 500 μL of pre-warmed Zymo Elution Buffer (10mM Tris, 0.1mM EDTA, pH 8.5). An overview of methods can be found in **Table 1**; **Supplementary Figure 1**, and full details of each method are available in **Supplementary File 1**. Extracted DNA was assessed for concentration and purity using a NanoDrop 2000c spectrophotometer (Thermo Fisher Scientific, Carlsbad, CA U SA). Once the most efficient method was determined based on quantity of DNA, fragment size, and ability to be classified, that method was presented in the FF versus FT comparison.

**Table 1:** Overview of extraction methods.

Method	Abbreviation	Treatment 1	Treatment 2	Treatment 3	DNA pull down method
No bead beating	0BB	Lysozyme -> SD S + ProtK	N/A	N/A	Magnetic Beads
3 minutes bead beating	3BB	Bead beat for 3 minutes	Lysozyme -> SD S + ProtK	N/A	Magnetic Beads
Double digestion and Zymo	DDZ	Lysozyme -> SD S + ProtK	Bead beat for 3 minutes	Lysozyme -> SD S + ProtK	Magnetic Beads
Double digestion and precipitation	PREC	Lysozyme -> SD S + ProtK	Bead beat for 3 minutes	Lysozyme -> SD S + ProtK	Ethanol precipitation

### 2.5 Sequencing

Sequencing libraries were prepared using the Native barcoding kit ( SQK-NBD114.96, Oxford nanopore Technologies, Oxford UK) and sequenced using PromethION flow cells (R10.4.1) on a P2 Solo device attached to a GridION (ONT) with MinKnow v 22.08.9 and super accurate base calling mode selected along with barcode trimming. All other parameters were set as default. Samples were basecalled on the machine with the most up to date Dorado basecaller.

### 2.6 Bioinformatics processing

Raw reads were initially processed with Porechop (v0.2.4) (https://github.com/rrwick/Porechop) to trim sequencing adapters and residual barcodes. Following adapter removal, reads shorter than 300 nucleotides were filtered out to ensure high-quality downstream analyses. Quality metrics, including total read count, N50, and quality score distributions, were evaluated using Nano Stat (v1.6.0) on both raw reads and the filtered reads (De Coster et al., 2018). A summary of this data and the percent lost from each method, and a breakdown of the number of reads by fragment size, can be found in supplemental sheets. For taxonomic classification, filtered reads were analyzed using Kaiju (v1.9.2) with the nr_euk reference database, operating in mem mode and a maximum match length of 12 (-m 12).

### 2.7 Data analysis

To assess differences in read length across experimental conditions, we employed non-parametric rank-based tests appropriate for the non-normal distribution of the data. For comparisons among the four DNA extraction methods (0BB, 3BB, DDZ, and PREC), we used a Kruskal-Wallis rank sum test to test for overall group differences in read length. When significant differences were detected, we performed Dunn’s *post hoc* pairwise comparisons using the F SA R package (v0.10.0), with Benjamini-Hochberg correction applied to control the false discovery rate.

To evaluate the effect of preservation method FF vs. FT on read length a Kruskal-Wallis test was applied. Differences in taxonomic composition were assessed at the domain level (Bacteria, Archaea, Eukaryota, and Other) by calculating the percentage of classified reads per sample. To determine whether archaeal abundance differed by preservation method, we used a paired Wilcoxon signed-rank test comparing archaeal read proportions between FF and FT treatments within individual cows.

Finally, to examine whether preservation method had differential impacts on Gram-positive vs. Gram-negative bacteria, we categorized reads accordingly using a curated classification table, and performed a Kruskal-Wallis test across the four treatment/classification groups (Gram-Positive.FF, Gram-Positive.FT, Gram-Negative.FF, Gram-Negative.FT). Significant results were followed by Dunn’s test with Benjamini-Hochberg correction. All visualizations, including boxplots and stacked bar plots, were generated using the ggplot2 R package.

## 3 Results

### 3.1 Simulated reads

To determine the influence of read length on taxonomic classification, we simulated sequencing reads from 22 genomes commonly found in the rumen microbiome, including both bacterial and archaeal taxa. Each synthetic sample was classified using Kaiju parameters identical to those applied in the experimental dataset.

Classification outcomes showed a consistent trend: longer reads were more likely to be classified by Kaiju (**Figure 1**). Misclassification rates were highest among shorter reads and declined progressively with length, while the proportion of correctly classified reads increased (**Supplementary Figure 2**). This result highlights a key limitation of short-read metagenomics in taxonomic resolution, particularly for genomes with limited representation in reference databases.

**Figure 1:**
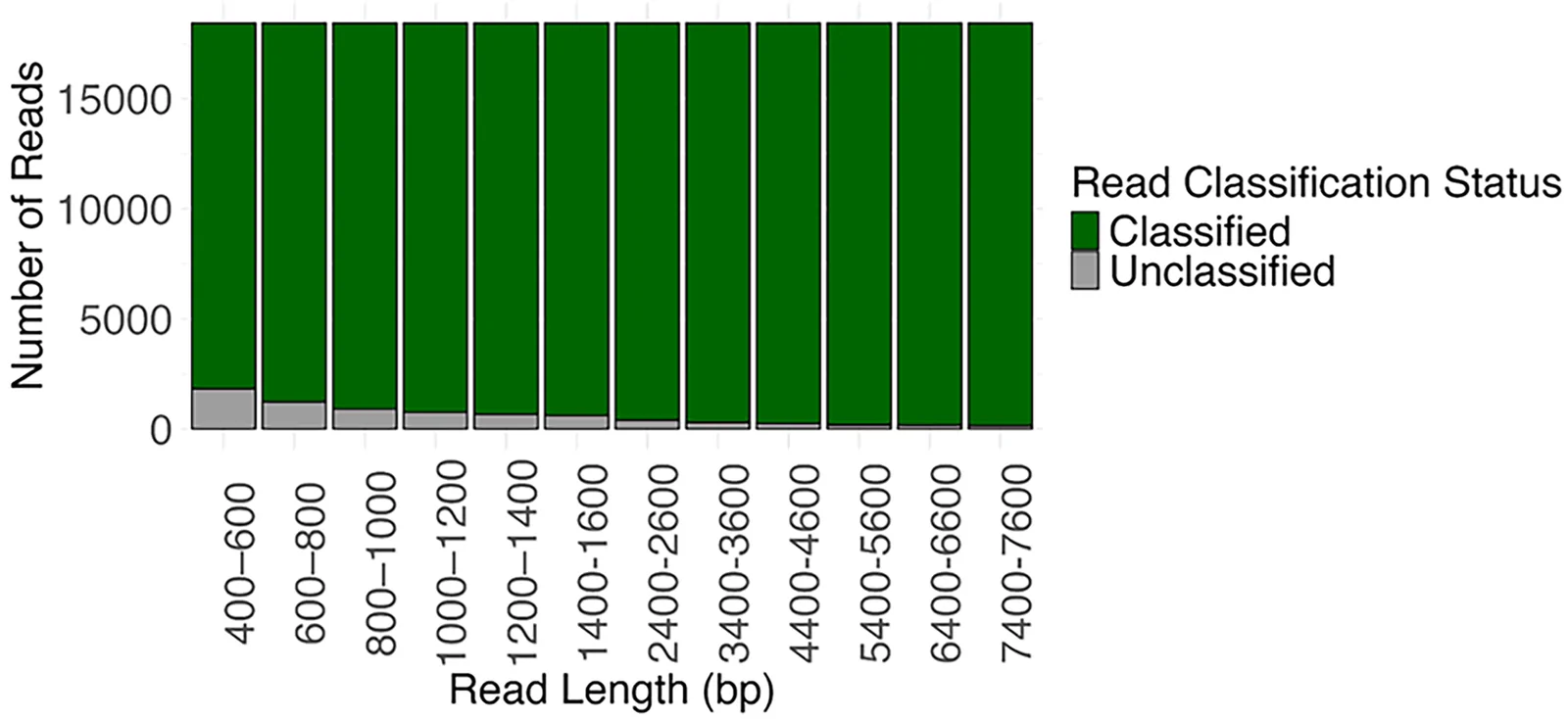
Number of synthetically generated reads classified by Kaiju increases with read length. Stacked bar graphs indicating the number of reads Classified (green) and Unclassified (grey) by Kaiju. Read length windows are shown on the X axis.

Notably, many of the observed “misclassifications” stemmed from minor discrepancies in naming conventions or taxonomic labels across databases (**Supplementary Figure 3**). However, true misclassification remained common among archaeal reads, largely due to redundancy and poor representation of archaeal genomes in the reference database (**Supplementary Figure 4**). This reinforces the challenge of recovering archaeal diversity in rumen metagenomic studies and indicates a need for more curated reference datasets.

Given the profound effect of read length on correct taxonomic classification, we compared two sample preservation techniques paired with four extraction techniques to determine which combination of methodologies would be most appropriate for Oxford Nanopore sequencing.

### 3.2 DNA yield and quantity

The precipitation method provided the most DNA out of all the methods tested (**Table 2**). It also had the highest average 260:230 ratio. On average, it had the highest 260:280 ratio when performed on the FT samples and the second highest ratio when performed on FF samples. Conversely, 0BB performed the worst out of all methods on yield followed by DDZ. The 260:230 ratio was lower than expected for all samples, although the FT 3BB sample averaged the lowest at 0.29 and the FF 0BB sample averaged 0.31.

**Table 2:** Quality and quantity of DNA from different extraction methods performed on FT samples and effect of FF on the PRECIP extraction method on Rumen liquid samples from 4 different cows.

Cow	Preservation methods	Extraction methods	Nucleic acid (ng/ul)	260:230	260:230	Total quantity (ug)
1	FT	0BB	30.2	1.27	0.18	2.3
2	FT	0BB	19.9	0.83	0.07	1.5
3	FT	0BB	18.6	1.79	0.8	1.4
4	FT	0BB	14.6	1.58	0.5	1.1
1	FT	3BB	69.6	1.47	0.23	5.2
2	FT	3BB	172.8	1.28	0.15	13.0
3	FT	3BB	43.1	1.62	0.35	3.2
4	FT	3BB	60.2	1.64	0.44	4.5
1	FT	DDZ	43.2	1.7	0.46	3.2
2	FT	DDZ	26.8	1.63	0.55	2.0
3	FT	DDZ	56.5	1.63	0.55	4.2
4	FT	DDZ	70.7	1.54	0.29	5.3
1	FT	PREC	611.9	1.71	0.64	458.9
2	FT	PREC	357.9	1.81	0.77	268.4
3	FT	PREC	1428.8	1.73	0.88	1071.6
4	FT	PREC	484.5	1.78	0.9	363.4
1	FF	PREC	527.4	1.53	0.53	395.6
2	FF	PREC	346	1.48	0.5	259.5
3	FF	PREC	631.8	1.74	0.94	473.9
4	FF	PREC	616.4	1.78	0.93	462.3

### 3.3 Read length and sequencing yield

#### 3.3.1 DNA extraction method performance

A Kruskal-Wallis rank sum test was conducted to examine differences in read length across the four extraction methods (0BB, 3BB, DDZ, and PREC). The average read length of each sample replicate, resulting in 24 samples per extraction method, was used to perform significance testing. The test indicated a statistically significant difference among the groups, *p=*9.98 X 10^-3^, suggesting that at least one group differed from the others. *Post hoc* pairwise comparisons using Dunn’s test with Benjamini-Hochberg correction identified a significant difference between 0BB and PREC (*p* = 4.72×10^−3^) (**Table 3**). No other pairwise comparisons were statistically significant after adjustment. These findings can be observed in **Figure 2A**, which shows higher median read lengths for both DDZ and PREC compared to 0BB. The distributions skew longer for DDZ and PREC, while 0BB has a narrower range of read lengths and a lower median. Additionally, **Figure 2B**, which displays the number of reads across extraction methods, shows that 0BB and 3BB tend to produce higher read counts, while DDZ and PREC result in lower total yields, suggesting a trade-off between read length and read count.

**Table 3:** Results of Dunn’s test following a significant Kruskal-Wallis test (p-value = 0.00047), comparing read lengths among four extraction methods (0BB, 3BB, DDZ, PREC).

Comparison	Z	p-value	Adjusted p-value (Benjamini-Hochberg)
0BB-DDZ	-1.751318845	0.078981003	0.159782007
0BB-3BB	-1.91712418	0.055222157	0.165666472
DDZ-3BB	-0.165805334	0.868310148	0.868310148
0BB-PREC	-3.357558023	0.000786342	**0.004718054**
DDZ-PREC	-1.606239178	0.108221368	0.162332053
3BB-PREC	-1.440433843	0.149744695	0.179693633

The table reports the unadjusted p-values, and Benjamini-Hochberg-adjusted p-values (P.adj) for each pairwise comparison. Statistically significant value in bold.

**Figure 2:**
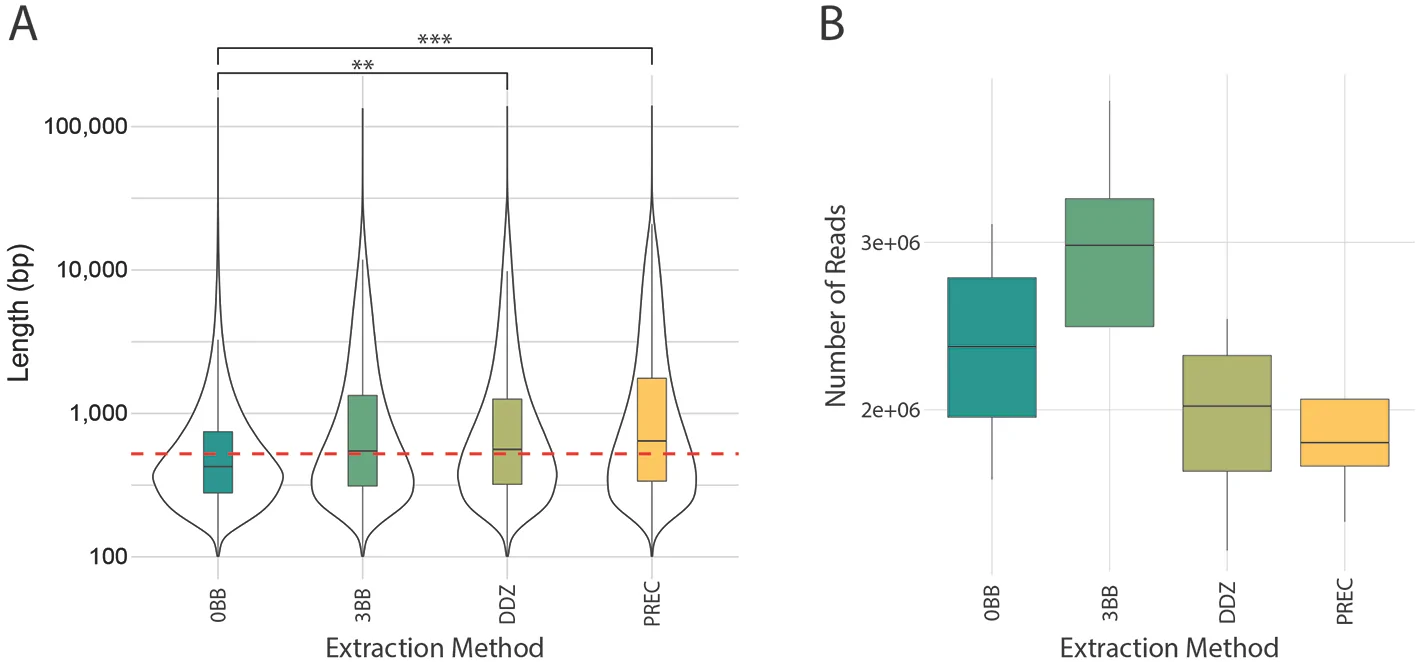
Read properties across extraction methods. **(A)** Read length for each extraction method are displayed as boxplots inside violin plots representing sample distribution. The dashed red line represents the grand median. **(B)** Boxplot showing the total number of sequencing reads obtained from each extraction method. All colored boxplots represent the median, upper and lower quartiles with whiskers extending to 1.5 x IQR.

#### 3.3.2 DNA preservation method performance

To assess the impact of preservation method on read length, a Wilcox test was performed comparing read lengths between FF and FT treatment. The analysis revealed a significant difference in read length distributions between the two groups (*p* = 1.73×10^−17^) (**Table 3**). As shown in **Figure 3A**, samples preserved via FF exhibited noticeably longer and more variable read lengths compared to those preserved by FT, which showed a narrower and shorter distribution. This suggests that FF preservation better maintains DNA integrity, resulting in longer sequencing reads, while FT may lead to increased fragmentation or degradation. The trend of preferential sequencing for shorter reads was corroborated when comparing the read counts between FF and FT, where many more reads were found in the FT group (**Figure 3B**).

**Figure 3:**
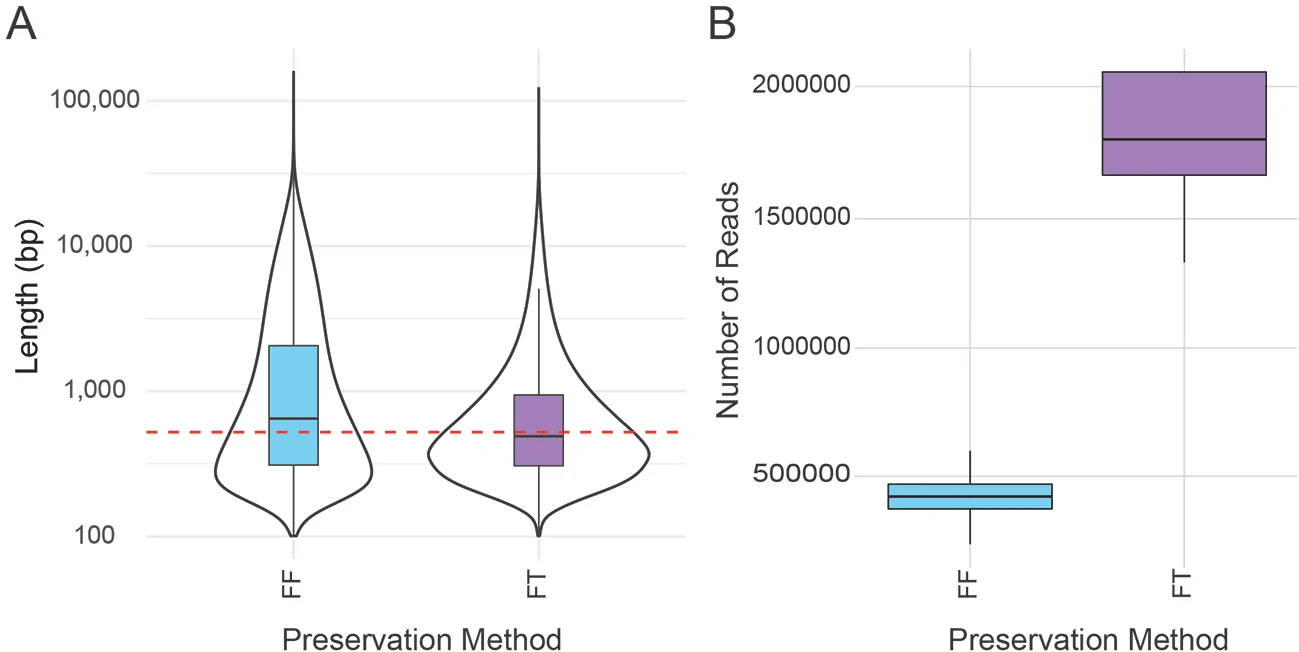
Read properties across preservation methods. **(A)** Read length for each preservation method are displayed as boxplots inside violin plots representing sample distribution. The dashed red line represents the grand median. **(B)** Boxplot showing the total number of sequencing reads obtained from each extraction method. All colored boxplots represent the median, upper and lower quartiles with whiskers extending to 1.5 x IQR.

### 3.4 Microbial community composition under different preservation methods

We further assessed the effect of preservation methods on the taxonomic composition of rumen microbial communities. **Figure 4** shows the proportional composition of reads assigned to each domain across four cows under the two preservation conditions (FF and FT). The bacterial fraction dominated all samples, followed by Eukaryotes and Archaea. While overall domain-level composition remained relatively stable between treatments, a decrease in the relative abundance of Archaea was observed in FT-treated samples compared to their FF counterparts. To evaluate this pattern, the proportion of reads classified as Archaea was quantified per sample using a paired Wilcoxon signed-rank test (**Figure 5**). The test revealed a significant decrease in Archaea abundance under FT conditions (*p* = 4.90×10^−4^). Within-individual comparisons highlight that in all cows, Archaea representation was reduced under FT treatment. These results indicate that preservation methods can introduce bias in microbial community profiling, particularly impacting the recovery or detectability of archaeal DNA, suggesting that FT may differentially degrade archaeal DNA.

**Figure 4:**
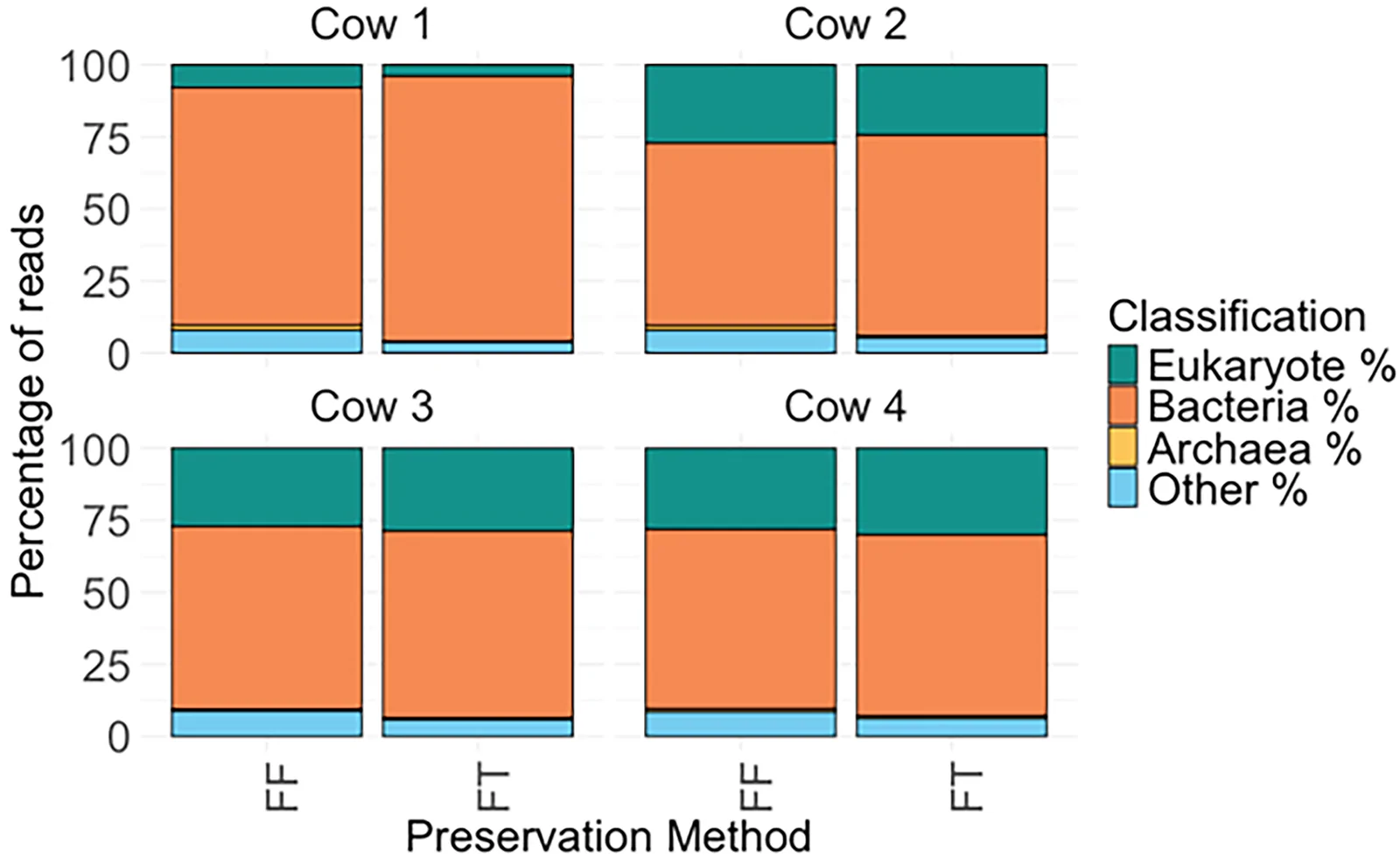
Percentage of reads belonging to major microbial domains by preservation method across individual cows. Stacked bar plots showing the percentage of reads classified as Eukaryota, Bacteria, Archaea, and Other across four cows under two preservation methods: Flash-Freeze (FF) and freeze-thaw (FT). Each bar represents one sample from a cow preserved using the PREC protocol.

**Figure 5:**
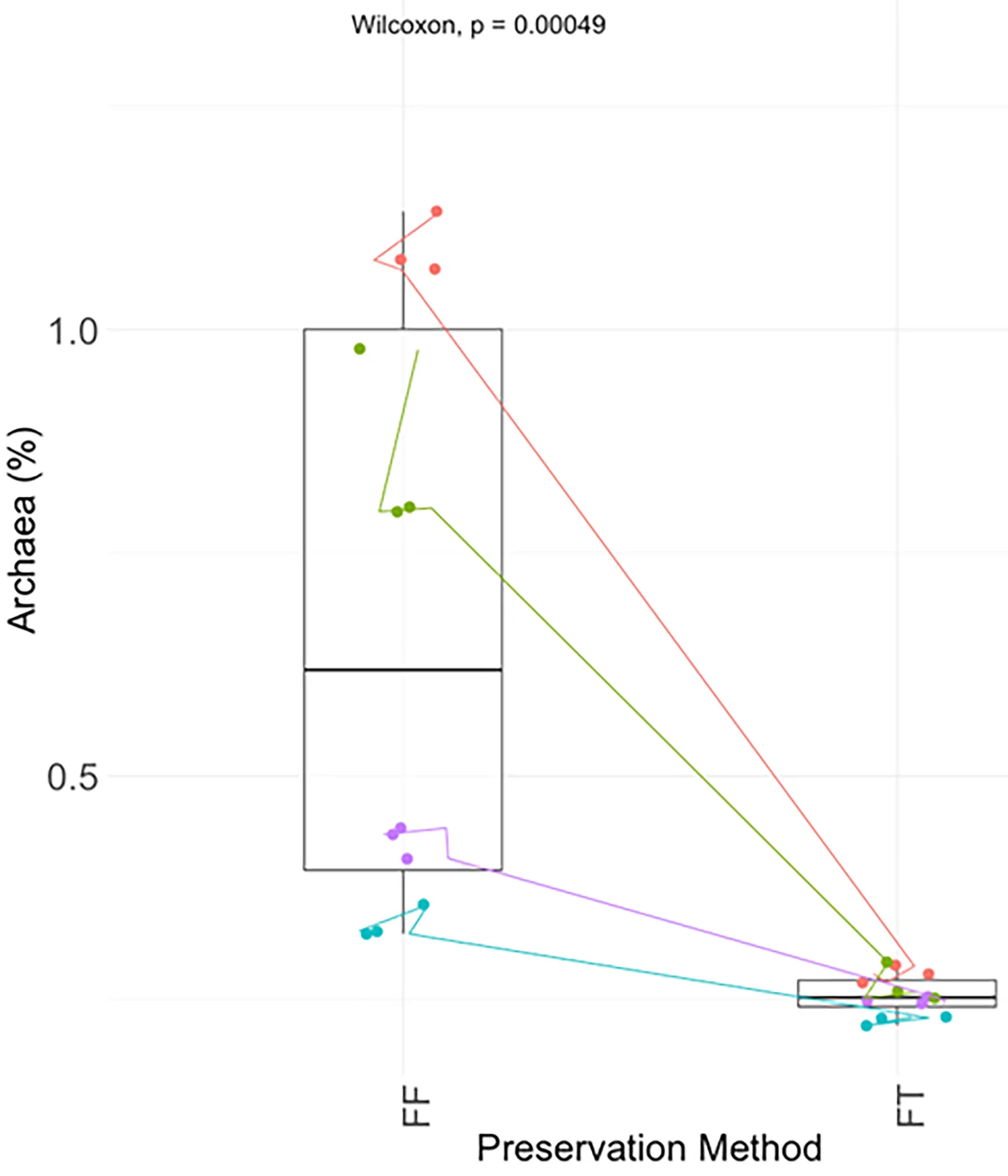
Archaeal abundance significantly decreases under freeze-thaw preservation. Boxplots indicating the mean, upper and lower quartiles of the proportion of archaeal reads under Flash-Freeze (FF) and freeze-thaw (FT) conditions with whiskers extending to 1.5 x IQR. Each point represents a sample, with lines connecting paired samples from the same cow.

### 3.5 Differential impact on gram-positive vs. gram-negative bacteria

To determine whether preservation method FF vs. FT differentially affects read length in Gram-positive versus Gram-negative bacteria; we performed a Kruskal-Wallis rank sum test across the four treatment–classification combinations. The test revealed a statistically significant difference in read length distributions among the groups (p <1.00 X 10^-3^, **Figure 6**). *Post hoc* pairwise comparisons using Dunn’s test with Benjamini-Hochberg correction (**Table 4**) indicated that FF sample collection resulted in significantly longer reads than FT when comparing both Gram-Negative (vs FT Gram-Neg. *p_adj_* = 1.61×10^−4^, vs FT Gram-Pos. *p_adj_*= 2.71×10^−6^) or Gram-Positive (vs FT Gram-Pos. *p_adj_* = 5.64×10^−4^, vs FT Gram-Neg. *p_adj_* = 1.30×10^−2^). No significant difference was identified when comparing Gram-Positive to Gram-Negative read lengths for FF (*p* = 0.19) or FT (*p* = 0.34). This interpretation is supported by the read length distributions in **Figure 7**, where FF samples consistently show higher medians and longer upper tails across both bacterial classes. Furthermore, **Figure 6** displays an increase in Gram-positive counts per million (CPM) in FF samples compared to FT within each cow (Wilcoxon test, *p* = 5.83×10^−12^).

**Table 4:** Results of Dunn’s test following a significant Kruskal-Wallis test (p-value = 4.245e-07), comparing read lengths among the combination of treatment and classification (Gram-Positive.FT, Gram-Negative.FT, Gram-Positive.FF, Gram-Negative.FF).

Comparison	p-value	Adjusted p-value (Benjamini-Hochberg)
Gram-Negative.FF - Gram-Negative.FT	5.358815e-05	1.607645e-04
Gram-Negative.FF - Gram-Positive.FF	1.572096e-01	1.886515e-01
Gram-Negative.FT - Gram-Positive.FF	8.667932e-03	1.300190e-02
Gram-Negative.FF- Gram-Positive.FT	4.520907e-07	2.712544e-06
Gram-Negative.FT - Gram-Positive.FT	3.143173e-01	3.143173e-01
Gram-Positive.FF - Gram-Positive.FT	2.822322e-04	5.644643e-04

The table reports the unadjusted p-values, and Benjamini-Hochberg-adjusted p-values (P.adj) for each pairwise comparison.

**Figure 6:**
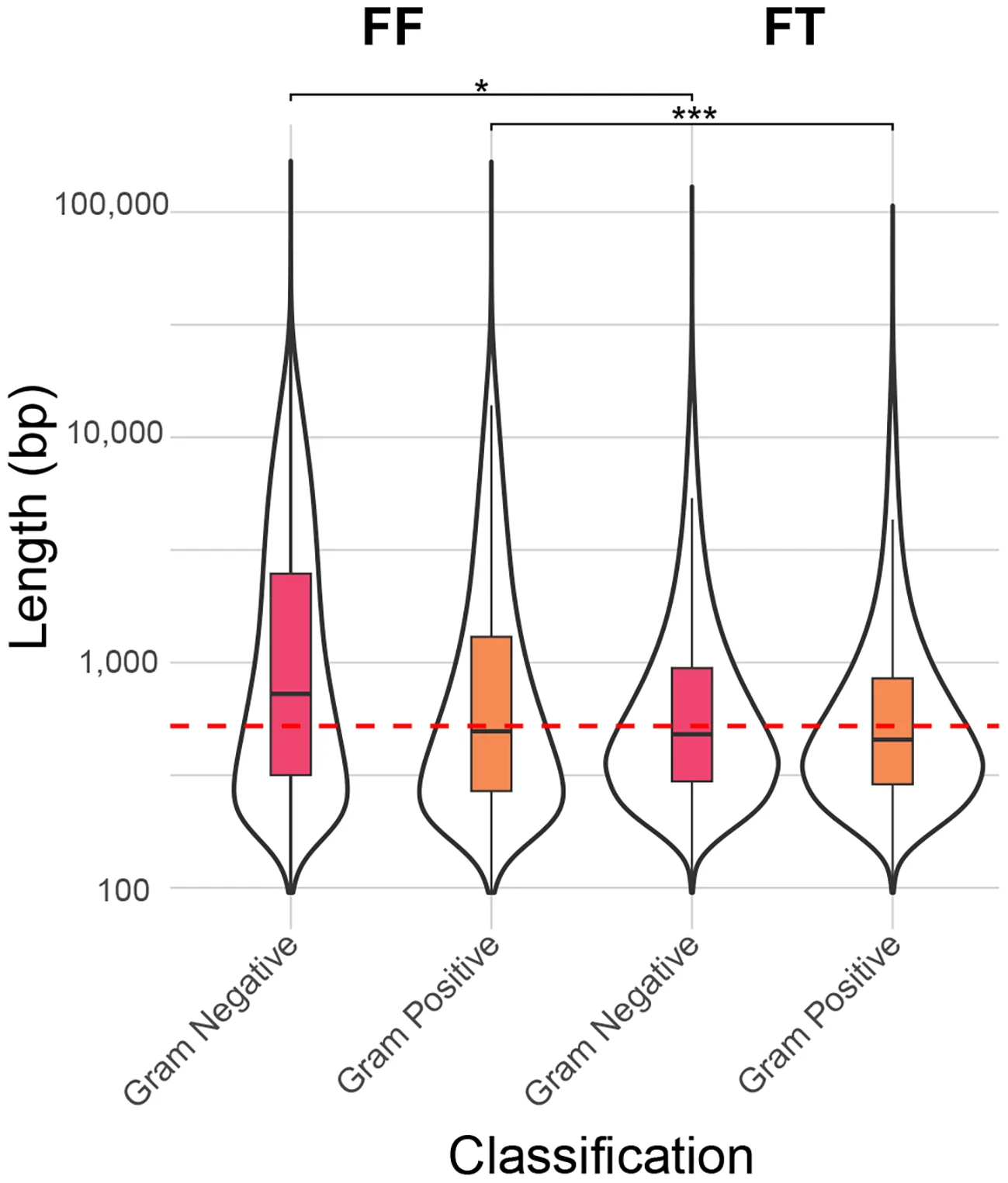
Read length distribution across preservation methods and bacteria classification. Boxplots displaying read lengths of gram-negative (pink) and gram-positive (orange) read lengths of samples Flash-frozen (FF) or Freeze-thawed (FT) with distributions displayed as violin plots. Boxplots are median centered with colored bounds extending to the upper and lower quartiles and whiskers to 1.5 x IQR. Significance thresholds are indicated by asterisks with *(p < 0.05), **(p < 0.01) and ***(p < 0.001).

**Figure 7:**
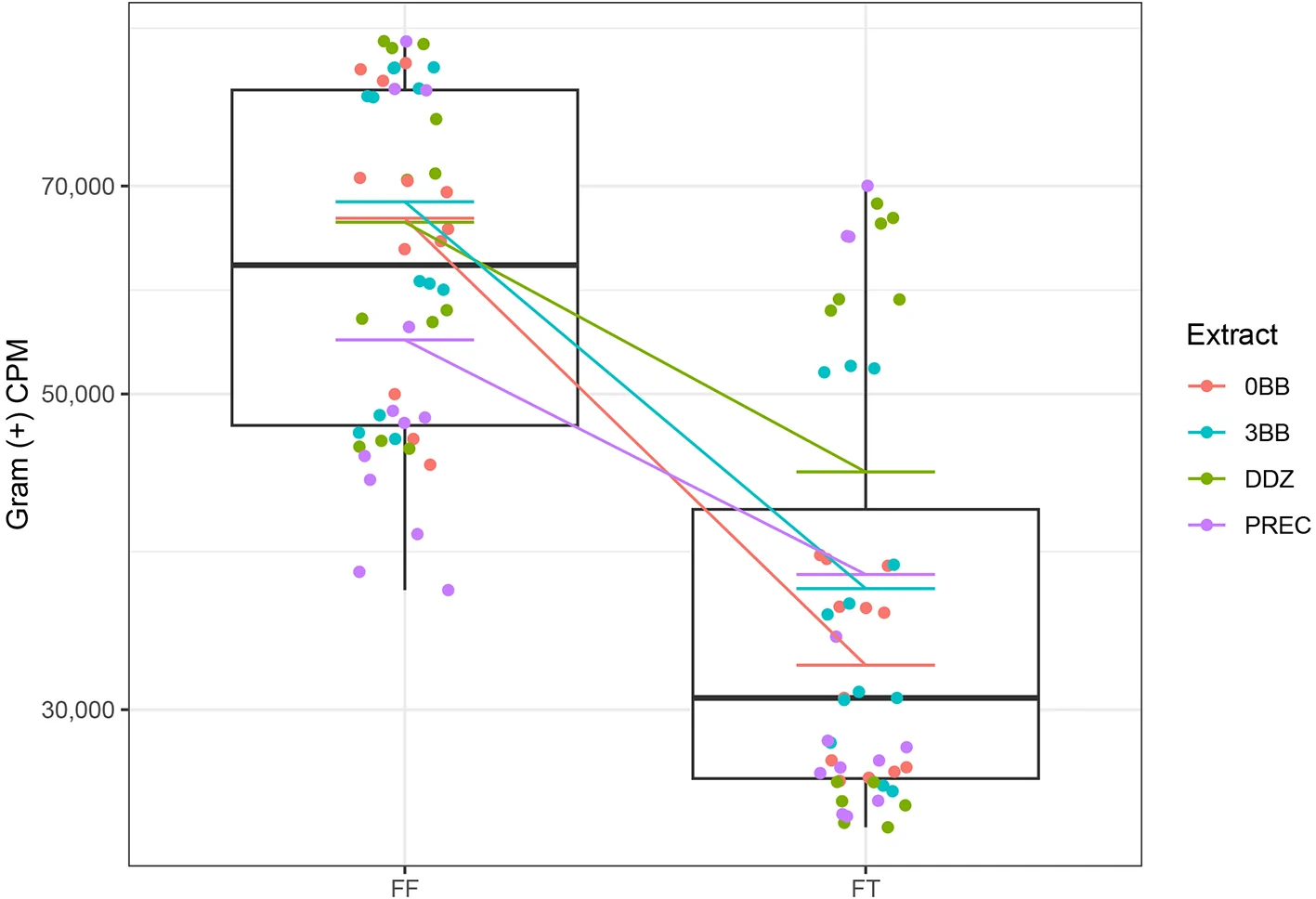
Counts per million comparison of gram-positive reads in flash-frozen and freeze-thawed samples. Boxplots indicating the mean, upper and lower quartiles of gram-positive reads are displayed from Flash-Freeze (FF) and freeze-thaw (FT) conditions are displayed. Colored points indicate the extraction method with each extraction method’s grand median displayed as a colored line.

## 4 Discussion

Long-read sequencing technologies have greatly enhanced our ability to assemble complex genomes and extract functional insights from microbial communities (Kim et al, 2024). However, the quality and representativeness of these data are strongly influenced by upstream processing, particularly sample preservation and DNA extraction methods. Previous studies utilized bulk freezing which we have determined can change the bacterial composition (Henderson et al., 2013; Mott et al., 2022; Xie et al., 2022 (**Figure 7**; **Supplementary Figure 2**). We hypothesize this strategy is utilized because researchers often do not have the time to fully extract large quantities of samples after a full day of sample collection. With this in mind, we developed the FF processing method to reduce the osmotic pressure and natural enzymatic digestion of microbes in the collected liquid that occurs when thawing bulk samples. Despite the importance of sample handling and processing, few studies have systematically examined how these steps affect both DNA quality and microbial representation from complex samples, preferring to rely on mock communities (Signorelli et al., 2026, and Mott et al., 2022). McCann et al. (2014) do not even mention how DNA extraction or collection method can affect the data from high-throughput sequencing in their review. Furthermore, current literature centers on the effect of preservation or extraction method using 16 S or other amplicon sequencing, not long read sequencing technologies (Henderson et al., 2013; Johnson et al., 2025; Mott et al., 2022; Poulsen et al., 2021; Huws et al., 2018). Using experimental and simulated datasets, we determined that shorter reads from suboptimal handling and sample preparation disproportionately impact archaeal and Gram-negative taxa, thereby reducing taxonomic accuracy and affecting overarching conclusions about community composition. Our results indicate the need to optimize preservation and extraction protocols to maintain biologically meaningful variation and ensure metagenomic profiles accurately reflect true community composition. In this study, we specifically evaluated how processing and extraction procedures influence read length, sequencing yield, and taxonomic composition in the rumen microbiome.

To interrogate the relationship between read length and taxonomic classification accuracy, we generated synthetic reads ranging from 400 to 7,600 bp, the point at which previous lab data indicated a greater than 90% reduction in overall read counts, in 200 bp bins, using 22 representative rumen microbial genomes. This simulation elucidated that longer reads increased the likelihood of correct classification and reduced the proportion of unclassified or misclassified reads (**Figure 1**; **Supplementary Figures 3**–**5**), a trend consistent across diverse rumen taxa, including both bacteria and archaea. Importantly, these results suggest that the observed reduction in archaeal representation under FT conditions may not solely reflect actual loss of cells but also reduced detection power due to DNA fragmentation. True misclassification rates were notably higher for archaeal reads, likely due to redundancy and incomplete representation of archaeal genomes in reference databases limiting accurate classification, even for high-quality reads. Overall, the simulated data analysis emphasizes that upstream preservation and extraction strategies critically shape the apparent composition of the rumen microbiome by influencing both DNA integrity and our ability to detect and correctly classify taxa.

Our first experimental objective was to determine which extraction methods recover high-molecular-weight (HMW) DNA suitable for long-read sequencing. Across the four tested methods (0BB, 3BB, DDZ, and PREC), we observed significant differences in read length distributions (*p* = 9.98×10^−3^). Specifically, PREC yielded longer reads than 0BB, although they generated fewer total reads (**Figure 2B**). This result isn’t surprising given that shorter fragments will move quicker through Nanopores than the longer fragments. These methods incorporate steps that minimize mechanical shear and preserve DNA integrity during extraction. This emphasizes the need to optimize extraction protocols not just for yield, but for DNA fragment size and quality. Our results indicate that while 0BB, 3BB and DDZ protocols yielded less DNA, due to a magnetic bead-based purification step used to isolate only HMW DNA (**Table 2**), the use of magnetic beads significantly impacted overall DNA yield. Increasing the bead-to-sample ratio may mitigate this loss, though the manufacturer recommends a maximum of 50 µL of beads, which can bind up to 10 µg of DNA. This was additionally corroborated by fragment analysis (**Supplementary Figure 6**). The precipitation method produced far higher DNA yields, which is particularly advantageous when downstream size selection, performed via AMPure beads or agarose gel, is required. These methods of size selection are often avoided due to significant sample loss; however, with precipitation-based extraction, the starting yield is more than sufficient to accommodate loss regardless of storage method. Oral swabs have been proposed as a non-invasive method to capture rumen and oral microbes from ruminants (Kittelmann et al., 2015; Skarlupka et al., 2024). This method has often been relegated to PCR methods due to low yield (Mott et al., 2022). The PREC method was tested on swab samples and provided higher yields than traditional kits used for extraction (**Supplementary File 1**; Mott et al., 2022). The protocol proved equally effective in this context, recovering DNA in quantities suitable for Nanopore long-read whole-genome sequencing and suggesting broad applicability to sample types where DNA yield is a limiting concern. The method can be found in **Supplementary File 1** with the other protocols described in this manuscript.

We also evaluated how preservation strategies impact yield (**Table 2**), read length, and microbial composition. While the volume of magnetic beads used in 0BB, 3BB and DDZ was greater than the manufacture’s recommendation, it may have affected the amount of DNA yield. We were cautioned by the company not to use more than 40ul of beads as it would greatly affect the size selection of the assays. To try to maximize the amount of DNA from the beads, we eluted the DNA in 2 steps, heated the samples to 55°C, and increased the elution volume to 75ul to increase the likelihood that all DNA was eluted from the beads.

Method of sample preservation and time spent in storage has been established as having a significant effect on microbial populations (Granja- Salcedo et al., 2017; Johnson et al., 2025), though both studies relied on small fragments of the 16 S gene to draw their conclusion. A more recent study concluded that greater microbial diversity was associated with pelleting rumen fluid prior to freezing (Martinez-Fernandez et al., 2019). Another study concluded preservation with PB S-glycerol to be the optimal storage media for representative ruminal microbiomes (Fliegerova et al., 2014). However, these studies were limited to amplicon sequencing, usually the V4 region of the 16 S rRNA gene, not full scale metagenomic sequencing. As long read sequencing technologies become more affordable, enabling large scale metagenomic analyses, the effect of preservation technique on DNA length is a pertinent concern for ruminal microbiologists. In the present study flash freezing preserved DNA integrity significantly better than freeze-thaw treatments, resulting in longer reads (p = 1.73×10^−17^, **Figure 3A**).

Recovery of archaeal DNA is affected by sample preservation method (McKain et al., 2013) with samples stored in glycerol significantly reducing yields. In the present study, which did not include a cryoprotectant, FF samples also maintained higher archaeal abundance (**Figure 5**), while FT led to a significant drop in archaeal reads (p = 4.9×10^−4^), suggesting that these cells or their DNA may be more susceptible to degradation. While flash freezing is preferential to using a cryoprotectant for archaea, these data point to bulk freezing followed by thawing as significantly affecting archaeal DNA recovery.

Furthermore, regardless of gram stain status, FF produced longer reads. These results were statistically significant compared to FT samples (**Table 3**; **Figure 5**). We chose to focus on Gram-negative and Gram-positive bacteria due to their known cell wall structures which serve as a proxy for the severity of each treatment due to Gram-positive bacteria being more recalcitrant to enzymatic, mechanical, and osmotic disruption than Gram-negative bacteria. Gram-negative reads were shorter in FT-treated samples, supporting the hypothesis that Gram-negative bacteria may be more susceptible to degradation during freeze-thaw cycles due to their more fragile outer membrane ( Supplementary Sheets). There was a 56-82% difference in average gram-negative read length on raw reads, and between 63-79% difference when all reads below 300bp were removed from the data set (**Supplementary File 1**). We also observed Gram-positive reads to be better preserved under FF, with greater normalized read counts of Gram-positive reads recovered (*p* = 5.83×10^−12^, **Figure 7**; **Supplementary File 1**) reinforcing the importance of sample preservation technique for accurate community profiling. As many historical experiments utilize bulk freezing, this data could impact the results of those studies as bulk freezing would introduce a bias towards gram-positive bacteria and other more resilient microbes.

Altogether, these results underscore the critical importance of thoughtful sample handling in long-read metagenomic studies. FF preservation combined with PREC extraction maximizes read length while preserving taxonomic representation and produces a large quantity of high-quality DNA, offering a best-practice strategy for future rumen microbiome studies. Conversely, suboptimal preservation or extraction can fragment DNA beyond recognition, biasing metagenomic profiles even when overall community composition appears unaltered. Beyond taxonomic accuracy, these effects have implications for interpreting rumen microbial function: underrepresentation of Archaea or Gram-negative bacteria may distort assessments of key microbial interactions and metabolic potential, as these groups are central to fermentation networks and nutrient turnover. Reduced recovery could underestimate the ruminal community’s digestive capacity. Together, these findings highlight that sample handling not only affects DNA quality and read length but also shapes downstream inferences about the structure and function of the rumen microbiome.

## 5 Conclusion

The presented results emphasize the role of choice of sample protocols in long-read metagenomic analysis. Both DNA extraction and preservation methods introduce tradeoffs that impact read quality, quantity and taxonomic annotation. Importantly, DDZ and PREC extraction methods are optimal for recovering HMW DNA but yield fewer total reads. Flash-freezing significantly preserves read length and prevents taxonomic distortion compared to freeze-thaw. Choice of preservation method disproportionately affects Archaea and Gram-negative bacteria, potentially impacting functional interpretations. These insights are critical for improving genome recovery and for ensuring accurate taxonomy annotation in rumen microbiomes.

## Data Availability

The datasets presented in this study can be found in online repositories. The names of the repository/repositories and accession number(s) can be found below: https://www.ncbi.nlm.nih.gov/, PRJNA1337107.

## Supplementary material

Supplementary Figure 1 Diagram-of experimental design. Colored boxes and outlines correspond to identical steps within the procedures.

Supplementary Figure 2 Percent gram-positive, gram-negative, eukaryote, unclassified, and other reads for flash-frozen versus freeze-thawed samples. Box plots display the percent of gram-positive, gram-negative, eurkaryota, unclassified, and other reads. FF samples are in pink and the FT samples are in teal. The X axis displays the extraction methods while the Y axis displays the percent reads. Each colored dot represents an individual cow. Statistics above each plot indicate Benjamin-Hochberg (p-adj-BH<0.05) statistics for each category. Anything that was not classified as gram-positive or negative, eukaryote, or unclassified were placed in the other category. This includes but is not limited to any bacteria that were not classified as gram-negative or positive, viruses, or archaea.

Supplementary Figure 3 Degree of misclassification is reduced by extending read length. Stacked bar graphs display the percentage of correctly classified (green), misclassified (red) and unclassified (grey) synthetic reads. Read length windows are displayed on the X axis.

Supplementary Figure 4 Number of misclassified reads by organism. The number of misclassified reads are sorted by the true organism (y-axis) and displayed as red bar counts across the x-axis.

Supplementary Figure 5 Reads Determined to be Misclassified by Assigned and True Genus. Misclassified reads for the True Organism (y-axis) are displayed bars (x-axis) colored by assigned organism. The majority of misclassifications stem from closely related species.

Supplementary Figure 6 Comparison of read percentages by preservation and extraction method. Read percentages were determined for each bin (displayed across the X-axis) for samples ran on a Femtopulse fragment analyzer (red) and from dereplicated Nanopore reads (blue). Femtopulse data was run 2 years after sequencing data once a machine was purchased. Box plots display the median and upper and lower quartiles with whiskers extending to 1.5 x IQR.

Supplementary File 1 Extraction protocols. All protocols mentioned in this manuscript have been included to lend clarity to the method section
