# Supplementary material for: Impact of rumen microbiome sample handling and extraction procedures on downstream long-read sequencing
Source: Front Cell Infect Microbiol. 2026 Oct 28;16:1907783. doi: 10.3389/fcimb.2026.1907783 (PMC13751430; doi:10.3389/fcimb.2026.1907783)
Supplement: Supplementary file 1 [file SupplementaryFile1.pdf]

Zymogen Quick-DNA HMW MagBead Kit without bead beating (0BB method)

McClure Lab -2-8-22

Reagents:
Zymo DNA/RNA shield- catalog number: R1200-125
Zymo Quick-DNA HMW MagBead kit (D6060)
Zymo Elution buffer-catalog number D30004-4-50 (10mM Tris, 0.1mM EDTA, pH 8.5)
Thermo Scientifif Proteinase K- catalog number: FERO0492
Sigma Lysozyme- catalog number (from Fisher): 50-177-9959

1.) Resuspend cell pellet in an equal volume of **DNA/RNA shield**
    a. Use at least 100ul of DNA/RNA shield, so if your pellet is 50ul still put in 100ul of **DNA/RNA shield**
2.) Gently pipette to resuspend
    a. only pipette as much as you have to, and don't be too vigorous
3.) Centrifuge sample 8000 xg for 1 minute
4.) Remove supernant and place in another tube
5.) Add 100ul of **PBS** to <u>PELLET</u> and pipette gently/stir to resuspend pellet
6.) Centrifuge 5000 xg for 1 minute
7.) Remove supernant and add it to the 1st sup tube
8.) Add 100ul of **TE** + 30ul of **Lysozyme** (100mg/ml) and pipette gently/stir to resuspend
9.) Incubate for 30 minutes at 37°C
10.) Turn the shaking hot block up to 55°C and place **Elution buffer** in block
11.) Add supernant back to sample
    a. If sup + sample is greater than 400ul split into ~400ul aliquots
12.) Add 30ul of **10% SDS** and 20ul of **ProtK** to sample, pipette gently/stir to mix
13.) Incubate at 55°C for 20 minutes
14.)Centrifuge 8000 xg for 1 minute to pellet residual debris
15.) Transfer supernant to a new low bind 1.5ml tube
16.) Add 2 volumes (~800ul) of **Quick –DNA Mag Binding Buffer** to sample and pipette to mix
17.) Shake the **MagBinding Beads** to put them into suspension
18.) Add 40ul of **MagBinding Beads** to your sample
19.)Place on hula mixer for 5 minutes
20.) Open tube lid and place on magnetic stand
21.) Once the beads have drawn to the magnet, remove supernant and discard
22.) Move sample off magnet and add 600ul of **DNA Pre-Wash Buffer**
23.) Pipette to resuspend and put on magnet stand with lid open
24.) Once the beads have drawn to the magnet, remove supernant and discard
25.) Move sample off magnet and add 900ul of **g-DNA Wash Buffer**
26.) Pipette to resuspend, transfer to a new tube, and put on magnet stand with lid open
27.) Once the beads have drawn to the magnet, remove supernant and discard
28.) Move sample off magnet and add 900ul of **g-DNA Wash Buffer**
29.) Pipette to resuspend and transfer to a new tube, put on magnet stand with lid open

30.) Once the beads have drawn to the magnet, remove supernant and discard

31.) Remove samples from magnet, do a quick spin in a microfuge, place back on the magnet, and carefully remove any residual **g-DNA wash buffer**

32.) Remove samples from magnet and place on 55°C heat block with lids open until beads are dry

  a. For many samples this takes between 5-10 minutes. If there is still residual g-DNA wash buffer, the samples will look shiny, if they are dry, they will look dull. DO NOT OVER DRY

33.) Add 40ul of warm Zymo elution buffer (EB) to the sample and resuspend the beads with gentle pipetting/stirring

34.) Place sample in 55°C hot block (with lid closed) for 10 minutes

35.) Transfer sample to magnet stand, and remove sup once beads have all settled near the magnet

  a. DO NOT Transfer any beads with your sample. To check this, place the tube with the supernant back on the magnet and look for any faint black lines

36.) Add another 35ul of warm EB to the beads, and resuspend gently

37.) Place the sample in 55°C hot block (with lid closed) for 10 minutes

38.) Transfer sample to magnet stand, and remove sup once beads have all settled near the magnet

39.) DNA is in the sup and is ready to be used!

Zymogen Quick-DNA HMW MagBead Kit with bead beating (3BB method)

McClure Lab-2-8-22

Reagents:
Zymo DNA/RNA shield- catalog number: R1200-125
Zymo Quick-DNA HMW MagBead kit (D6060)
Zymo Elution buffer-catalog number D30004-4-50 (10mM Tris, 0.1mM EDTA, pH 8.5)
Thermo Scientifif Proteinase K- catalog number: FERO0492
Sigma Lysozyme- catalog number (from Fisher): 50-177-9959
Biospec 0.1MM Zirconia/Sillica beads- catalog number: 11079101z
Biospec 0.5mm glass beads- catalog number: 11079105
Biospec Mini BeadBeater 24 disruptor- catalog number: 112011

1.) Resuspend cell pellet in an equal volume of **DNA/RNA shield**
    a. Use at least 100ul of DNA/RNA shield, so if your pellet is 50ul still put in 100ul of **DNA/RNA shield**
2.) Gently pipette to resuspend
    a. only pipette as much as you have to, and don't be too vigorous
3.) Place resuspended sample into a bead beating tube (2ml o-ring tube) with beads (0.3g of 0.1m beads and 0.1g of 0.5mm beads)
4.) Add samples to a Biospec Mini Bead Beater 24 and run at 3200 RPM for 3 minutes
5.) Centrifuge sample 8000 xg for 1 minute
6.) Remove supernant and place in another tube
7.) Add 100ul of **PBS** to PELLET and pipette gently/stir to resuspend pellet
8.) Centrifuge 5000 xg for 1 minute
9.) Remove supernant and add it to the 1st sup tube
10.) Add 100ul of **TE** + 30ul of **Lysozyme** (100mg/ml) and pipette gently/stir to resuspend
11.) Incubate for 30 minutes at 37°C
12.) Turn the shaking hot block up to 55°C and place **Elution buffer** in block
13.) Add supernant back to sample
    a. If sup + sample is greater than 400ul split into ~400ul aliquots
14.) Add 30ul of **10% SDS** and 20ul of **ProtK** to sample, pipette gently/stir to mix
15.) Incubate at 55°C for 20 minutes
16.) Centrifuge 8000 xg for 1 minute to pellet residual debris
17.) Transfer supernatant to a new low bind 1.5ml tube
18.) Add 2 volumes (~800ul) of **Quick –DNA Mag Binding Buffer** to sample and pipette to mix
19.) Shake the **MagBinding Beads** to put them into suspension
20.) Add 40ul of **MagBinding Beads** to your sample
21.) Place on hula mixer for 5 minutes
22.) Open tube lid and place on magnetic stand
23.) Once beads have drawn to the magnet, remove supernant and discard
24.) Move sample off magnet and add 600ul of **DNA Pre-Wash Buffer**
25.) Pipette to resuspend and put on magnet stand with lid open

26.) Once the beads have drawn to the magnet, remove supernant and discard
27.) Move sample off magnet and add 900ul of **g-DNA Wash Buffer**
28.) Pipette to resuspend, transfer to a new tube, and put on magnet stand with lid open
29.) Once the beads have drawn to the magnet, remove supernant and discard
30.) Move sample off magnet and add 900ul of **g-DNA Wash Buffer**
31.) Pipette to resuspend and transfer to a new tube, put on magnet stand with lid open
32.) Once the beads have drawn to the magnet, remove supernant and discard
33.) Remove samples from magnet, do a quick spin in a microfuge, place back on the magnet, and carefully remove any residual **g-DNA wash buffer**
34.) Remove samples from magnet and place on 55°C heat block with lids open until beads are dry
    a. For many samples this takes between 5-10 minutes. If there is still residual g-DNA wash buffer, the samples will look shiny, if they are dry, they will look dull. DO NOT OVER DRY
35.) Add 40ul of warm Zymo elution buffer (EB) to the sample and resuspend the beads with gentle pipetting/stirring
36.) Place sample in 55°C hot block (with lid closed) for 10 minutes
37.)Transfer sample to magnet stand, and remove sup once beads have all settled near the magnet
    a. DO NOT Transfer any beads with your sample. To check this, place the tube with the supernant back on the magnet and look for any faint black lines
38.)Add another 35ul of warm EB to the beads, and resuspend gently
39.)Place the sample in 55°C hot block (with lid closed) for 10 minutes
40.)Transfer sample to magnet stand, and remove sup once beads have all settled near the magnet
41.) DNA is in the sup and is ready to be used!

Modified Zymo Magbead method for Rumen pellets

(DDZ method)

McClure Lab 02-20-24

Reagents:
2X Zymo DNA/RNA shield- catalog number: R1200-125
Zymo Research MagBead binding Buffer- catalog number: D4077-1-250
Zymo Elution buffer-catalog number D30004-4-50 (10mM Tris, 0.1mM EDTA, pH 8.5)
Thermo Scientifif Proteinase K- catalog number: FERO0492
Sigma Lysozyme- catalog number (from Fisher): 50-177-9959
Biospec 0.1MM Zirconia/Sillica beads- catalog number: 11079101z
Biospec 0.5mm glass beads- catalog number: 11079105
Biospec Mini BeadBeater 24 disruptor- catalog number: 112011

1.) Label one 1.7ml tube with cow #, sample type, date collected, date extracted, DNA and initials for each
2.) Add an equal volume of 2x **DNA/RNA shield** to your sample (should be ~500ul-1ml)
a. Use at least 100ul of DNA/RNA shield, so if your pellet/sample is 50ul still put in 100ul of **DNA/RNA shield**
3.) Gently pipette to resuspend
a. only pipette as much as you have to, and don't be too vigorous
4.) Centrifuge sample 8000 xg for 1 minute
5.) Remove all supernant and place in another tube (called supernant tube now)
6.) Add 100ul of **PBS** to <u>PELLET</u> (original tube) and pipette gently/stir to resuspend pellet
7.) Centrifuge 8000 xg for 1 minute
8.) Remove supernant and add it to the supernant tube (will have 0.6 or 1.1ml in there now)
9.) Add 1ml of PBS, spin down and remove all liquid and discard
10.) Add 100ul of **TE** + 25ul of **Lysozyme** (100mg/ml) to the original tube and pipette gently/stir to resuspend
11.) Incubate for 30 minutes at 37°C
12.) After removing the tube from the hot block, turn the shaking hot block up to 55°C
13.) Add supernant from sup tube back to sample
14.) Add 40ul of **10% SDS** and 20ul of **ProtK** to sample, pipette gently/stir to mix
15.) Incubate at 55°C for 20 minutes
16.) Turn heat block back down to 37°C
17.) Centrifuge 8000 xg for 1 minute to pellet residual debris
18.) Transfer the liquid to a 1.5ml tube
    a. This is your lysed supernant tube
19.) Add 200 ul of TE to the sample tube and transfer resuspended pellet to a bead beating tube (2ml o-ring tube) with beads (0.3g of 0.1m beads and 0.1g of 0.5mm beads)
20.) Bead beat sample in a Biospec Mini Bead Beater 24 and run at 3200 RPM for 3 minutes
21.) Add 25 ul of lysozyme to sample tube and incubate for 30 minutes at 37°C
22.) Add 40ul of **10% SDS** and 20ul of **ProtK** to sample, pipette gently/stir to mix
23.) Incubate at 55°C for 20 minutes

24.) Centrifuge 8000 xg for 1 minute to pellet residual debris
25.) Transfer supernant to a 1.5ml tube
    a. This is your bead beaten supernant tube
26.) Add 2 volumes of Mag bead buffer to the lysed supernant tube and the bead beaten supernant tube
27.) Shake the **MagBinding Beads** to put them into suspension
28.) Add 40 ul of **MagBinding Beads** to each sample tube (not the one with beads)
29.) Place on hula mixer for 5 minutes
30.) Quick spin in minifuge
31.) Open tube lid and place on magnetic stand
32.) Once the beads have drawn to the magnet, remove supernant and discard
33.) Add 600ul of DNA Pre-Wash Buffer to each tube, and combine in a new tube
    a. At this point your Bead beaten supernant and lysed supernant are now in the same tube
34.) Move sample off magnet and add 600ul of **DNA Pre-Wash Buffer** and transfer to a new tube
35.) Pipette to resuspend and put on magnet stand with lid open
36.) Once the beads have drawn to the magnet, remove supernant and discard
37.) Move sample off magnet and add 900ul of **g-DNA Wash Buffer**
38.) Pipette to resuspend, transfer to a new tube, and put on magnet stand with lid open
39.) Once beads have drawn to the magnet, remove supernant and discard.
40.) Move sample off magnet and add 900ul of **g-DNA Wash Buffer**
41.) Pipette to resuspend and transfer to a new tube, put on magnet stand with lid open
42.) Once beads have drawn to the magnet, remove supernant and discard
43.) Remove samples from magnet, do a quick spin in a microfuge, place back on the magnet, and carefully remove any residual **g-DNA wash buffer**
44.) Remove samples from magnet and place on 55°C heat block with lids open until beads are dry
    a. For many samples this takes between 5-10 minutes. If there is still residual g-DNA wash buffer, the samples will look shiny, if they are dry, they will look dull. DO NOT OVER DRY
45.) Add 40ul of warm Zymo Elution buffer (EB) to the sample and resuspend the beads with gentle pipetting/stirring
46.) Place sample in 55°C hot block (with lid closed) for 10 minutes
47.) Transfer sample to magnet stand, and remove sup once beads have all settled near the magnet
    a. DO NOT Transfer any beads with your sample. To check this, place the tube with the supernant back on the magnet and look for any faint black lines
48.) Add another 35ul of warm EB to the beads, and resuspend gently
49.) Place the sample in 55°C hot block (with lid closed) for 10 minutes
50.) Transfer sample to magnet stand, and remove sup once beads have all settled near the magnet
51.) DNA is in the sup and is ready to be used!

Modified Zymo DNA precipitation method for Rumen pellets (PREC_method)

McClure Lab 05-08-24

Reagents:
2X Zymo DNA/RNA shield- catalog number: R1200-125
Zymo Research MagBead binding Buffer- catalog number: D4077-1-250
Zymo Elution buffer-catalog number D30004-4-50 (10mM Tris, 0.1mM EDTA, pH 8.5)
Thermo Scientifif Proteinase K- catalog number: FERO0492
Sigma Lysozyme- catalog number (from Fisher): 50-177-9959
Biospec 0.1MM Zirconia/Sillica beads- catalog number: 11079101z
Biospec 0.5mm glass beads- catalog number: 11079105
Biospec Mini BeadBeater 24 disruptor- catalog number: 112011

1. Label one 1.7ml tube with cow #, sample type, date collected, date extracted, DNA and initials for each
2. Add an equal volume of 2x **DNA/RNA shield** to your sample (most pellets are 100-200ul) and flick or pipette gently to mix
    a. Use at least 100ul of DNA/RNA shield, so if your pellet/sample is 50ul still put in 100ul of **DNA/RNA shield**
    b. You may need to use a pipette tip to dislodge the pellet from the tube, flick or using a pipette gently stir until the pellet is incorporated into the **DNA/RNA shield**
3. Place sample on Hula mixer and rotate end over end for 5 minutes
4. Centrifuge sample 8000 xg for 1 minute
5. Remove all supernant and place in a new 1.5ml tube (called "sup tube" now)
6. Add 100ul of **PBS** to PELLET (original tube) and pipette gently/stir to resuspend pellet
7. Centrifuge 8000 xg for 1 minute
8. Remove supernant and add it to the sup tube (may have 100-300ul in the sup tube)
9. Add 1ml of PBS,
10. Centrifuge 8000 xg for 1 minute
11. Remove all liquid without disturbing the pellet and discard
12. Add 100ul of **TE** + 25ul of **Lysozyme** (100mg/ml) to the original tube and pipette gently/stir to resuspend
13. Incubate for 30 minutes at 37°C in a hot block
14. After removing tube from hot block, turn the hot block up to 55°C
15. Add supernant from sup tube back to sample tube
16. Add 40ul of **10% SDS** and 20ul of **ProtK** to sample, flick or pipette gently/stir to mix
17. Incubate at 55°C for 20 minutes
18. Turn heat block back down to 37°C
19. Centrifuge 8000 xg for 1 minute to pellet residual debris

20. Transfer the supernant to a 15ml conical tube and set aside (room temperature is fine)
21. Add 200 ul of TE to the sample tube and resuspend the pellet
22. Transfer the resuspended pellet to a bead beating tube (2ml o-ring tube) with beads (0.3g of 0.1m beads and 0.1g of 0.5mm beads)
23. Bead beat sample in a Biospec Mini Bead Beater 24 and run at 3200 RPM for 3 minutes
24. Quick spin the tubes to pull any foam off of the cap
25. Add 25 ul of lysozyme (100mg/ml) to sample tube and incubate for 30 minutes at 37°C
26. Add 40ul of **10% SDS** and 20ul of **ProtK** to sample, flick or pipette gently/stir to mix
27. Incubate at 55°C for 20 minutes
28. Centrifuge 8000 xg for 1 minute to pellet residual debris
29. Add supernant to the 15ml tube from step 20 and discard the pelleted debris
    a. Be sure not to get any beads into your pipette while pulling off the supernant
30. Add 2 volumes of Mag bead buffer (MBB) to the tube
    a. i.e if you have 700ul of sample add 1,400ul of (MBB)
31. Add 3 (total volumes) of ethanol and invert multiple times
    a. i.e. from above you now have 2,100ul of sample +MBB so add 6,300ul of 100% ethanol
32. Spin 15ml conical tubes for 10' at 8,000 xg
33. The "pellet" may be more like a smear. Carefully dump supernant
34. Add 1ml of 70% ethanol and wash the smear off the side of the 15ml tube before transferring to a 1.5ml tube
    a. Sometimes the smear is tightly adhered to the tube so you can use your 1ml pipette tip to scrape it off into the 70% ethanol
35. Spin 1.5ml tube 17,000 x g for 5 minutes
36. Remove supernant and add 500ul of 70% ethanol to wash, flick or pipette gently/stir to mix
37. Spin sample for 17,000 x g for 1'
38. Removed supernant, quick spin, remove the rest of the ethanol
39. Dry the pellet (as best as you can ~5 minutes at 55°C)
    a. This helps evaporate any residual ethanol. Try not to over dry the pellet
40. Add 200ul-450ul of Zymo elution buffer (EB) to the sample to resuspend it and allowed it to sit at 55C for ~5 minutes.
    a. If the sample is still very chunky, you can add more EB to bring it into solution. If you have a very large pellet, it is not uncommon to need 1ml or more elution buffer to put it into suspension
    b. Sometime the liquid is cloudy due to so much DNA

Modified Zymo DNA precipitation method for Oral swabs

(McClure Lab 05-08-24)

Reagents:
Zymo DNA/RNA shield (2X)- catalog number: R1200-125
Zymo Research MagBead binding Buffer- catalog number: D4077-1-250
Zymo Elution buffer-catalog number D30004-4-50 (10mM Tris, 0.1mM EDTA, pH 8.5)
Thermo Scientific Proteinase K- FERO0492
Sigma Lysozyme- Catalog number (from Fisher)- 50-177-9959
TE- Tris EDTA pH 8.0
PBS- Phosphate Buffered Saline
Biospec Mini BeadBeater 24 disruptor- catalog number: 112011

NOTE: Oral swabs are collected in 2ml tubes with 0.5ml of PBS, so starting volume will be 0.5ml

1.) Label one 1.7ml tube with cow #, sample type, date collected, date extracted, DNA and initials for each
2.) Add 500ul 2x **DNA/RNA shield** to your sample (should be 1ml)
3.) Place sample on Hula mixer and rotate end over end for 5 minutes
4.) Centrifuge sample 8000 xg for 1 minute
5.) Remove all sup and place in another tube (called sup tube now)
6.) Add 100ul of **PBS** to original tube and pipette gently/stir to resuspend pellet
7.) Centrifuge 8000 xg for 1 minute
8.) Remove sup and add it to the sup tube (will have 1.1ml in there now)
9.) Add 1ml of PBS, spin down and remove all liquid and discard
10.) Add 100ul of **TE** + 25ul of **Lysozyme** (100mg/ml) to the original tube and pipette gently/stir to resuspend
11.) Incubate for 30 minutes at 37°C
12.) After removing tube from hot block, turn the shaking hot block up to 55°C
13.) Add 1.225ml of supernant from sup tube back to sample
14.) Add 40ul of **10% SDS** and 20ul of **ProtK** to sample, pipette gently/stir to mix
15.) Incubate at 55°C for 20 minutes
16.) Turn heat block back down to 37°C
17.) Centrifuge 8000 xg for 1 minute to pellet residual debris
18.) Transfer the liquid to a 50ml conical tube and set aside
19.) Add 200ul of TE to the sample tube
52.)Bead beat sample in a Bead beat sample in a Biospec Mini Bead Beater 24 and run at 3200 RPM for 3 minutes (no beads needed, just swab)
20.) Add 25ul of lysozyme to sample tube and incubate for 30 minutes at 37°C
21.) Add 40ul of **10% SDS** and 20ul of **ProtK** to sample, pipette gently/stir to mix
22.) Incubate at 55°C for 20 minutes
23.) Centrifuge 8000 xg for 1 minute to pellet residual debris
24.) Add supernant to 50 ml tube

25.) Add 2 volumes of Mag bead buffer (MBB)
   a. i.e. volume in 50ml conical tube should be 1,570ul therefore, you should add 3,140ul to the tube
26.) Add 3 total volumes of ethanol and invert
27.) i.e. from above you now have 6,280ul of sample +MBB so add 18,840ul of 100% ethanol
28.) Spin 50ml conical tubes for 10' at 8,000 xg
29.) The "pellet" may be more like a smear. Carefully dump supernant
30.) Add 1ml of 70% ethanol and wash the smear off the side of the 50 ml tube before moving it to a 1.5ml tube
31.) Spin 1.5ml tube 17,000 x g for 5 minutes
32.) Remove sup and add 500ul of 70% Ethanol to wash
33.) Spin sample for 17,000 x g for 1'
34.) Removed sup, quick spin, remove the rest of the ethanol
35.) Dry the pellet (as best as you can ~5 minutes at 55°C)
36.) Add 250ul of Zymo elution buffer (EB) to the sample to resuspend it and allowed it to sit at 55C for ~5 minutes. If the sample is still very chunky, you can add more EB to bring it into solution. Sometime the liquid is cloudy due to so much DNA
